# A suitable organic fertilizer substitution ratio could improve maize yield and soil fertility with low pollution risk

**DOI:** 10.3389/fpls.2022.988663

**Published:** 2022-09-08

**Authors:** Hao He, Mengwen Peng, Sibo Ru, Zhenan Hou, Junhua Li

**Affiliations:** ^1^Key Laboratory of Oasis Eco-Agriculture, Xinjiang Production and Construction Corps, College of Agriculture, Shihezi University, Shihezi, China; ^2^College of Life Sciences, Shihezi University, Shihezi, China

**Keywords:** scientific fertilization, crop growth, soil properties, heavy metals, sustainable agriculture

## Abstract

Organic fertilizer substitution (OFS) is an effective strategy for reducing the chemical fertilizer usage; however, the effects of different OFS ratios (OFSRs) on maize yield, soil fertility, and heavy metal pollution risk are still unclear. Therefore, determining a suitable OFSR is important. Through the pot experiment, no fertilizer (CK) and organic fertilizer substituting 0% (CF, chemical fertilizer alone), 8% (OF8), 16% (OF16), and 24% (OF24) of the chemical N fertilizer were set to investigate the effects of different OFSRs on maize growth and yield, soil properties (available nutrients, carbon fractions, and carbon pool indices), and nutrients and heavy metals in grain and soil. The results showed that OF8, OF16, and OF24 improved soil fertility by increasing soil organic carbon (SOC, by 10.05–16.26%) and its fractions, most middle- and micro-nutrients content, and carbon pool management index (CPMI, by 17.45–30.31%) compared with CF, while improving grain nutritional quality. However, they increased heavy metals content in grain and soil and their Nemerow comprehensive pollution index (NCPI, by 4.06–16.56% in grain and 2.55–5.57% in soil) but did not cause pollution. Among them, throughout the growth period, only OF8 treatment increased soil available nitrogen (AN), phosphorus (AP), and potassium (AK) content by 3.04–11.15%, 7.11–8.05%, and 0.12–6.05%, respectively, compared with CF, which thus significantly promoted maize growth and increased yield (by 35.65%); the NCPI of grain and soil was however lower than that OF16 and OF24. In conclusion, substitution ratio of 8% was considered ideal for promoting maize growth, improving yield and soil fertility, with a low pollution risk. The results of this study would aid in guiding the scientific application of OFS technology to agricultural production, thereby contributing to resource utilization of organic waste and sustainable agricultural development.

## Introduction

Over the last few decades, crop yields in China have increased by more than 40%, relying mainly on the use of chemical fertilizers (Ministry of Agriculture, 2015). In particular, the amount of chemical fertilizer applied to maize, a major grain and feed crop, accounts for 20.1% of the total chemical fertilizer applied to crops (Xu et al., 2019). Excessive application of chemical fertilizers in maize production to ensure yield has caused problems including increased planting costs, reduced soil fertility, and environmental pollution, limiting sustainable agricultural development (Guo et al., 2010; Zhao et al., 2016; Song et al., 2017; Lv et al., 2020). Therefore, scientific fertilization in agricultural production requires the consideration of crop yield and agricultural and environmental sustainability.

Organic fertilizer substitution technology plays an important role in reducing chemical fertilizer application and alleviating environmental pressures on agriculture (Liu et al., 2015; Song et al., 2017; Xin et al., 2017). Moreover, it ensures the supply of N, P, and K during crop growth, improving soil nutrient status because organic fertilizer supplements middle-nutrients (Ca, Mg, and S) and micro-nutrients (Mo, B, Fe, and Mn), which helps to increase crop yield (Osman, 2013; Redding et al., 2016; Ning et al., 2017; Yan et al., 2021). Additionally, OFS improves soil quality by increasing soil organic carbon (SOC) content (Manna et al., 2007; Saikia et al., 2015; Li J. et al., 2018). However, labile organic carbon (LOC) is sensitive to changes in soil C pool compared with SOC (Mandal et al., 2020), and can help in calculating the carbon pool management index (CPMI) (Loginow et al., 1987; Blair et al., 1995). Meanwhile, CPMI is a scientific indicator for assessing soil C pool and soil quality (Benbi et al., 2015; Duval et al., 2019; Saha et al., 2021; Zhang Y. R. et al., 2021). Studies have shown that OFS increases the SOC and LOC content, thereby improving the CPMI (Tang et al., 2020; Zhang et al., 2020). However, few studies have comprehensively analyzed the effects of OFS on crop yield, soil C fractions, CPMI, and soil nutrients.

Following China's “Zero Growth Strategy for Chemical Fertilizer” implementation, there has been an increasing demand for commercial organic fertilizers. Most commercial organic fertilizers are made from agricultural wastes (livestock manure and crop straw) through decomposition, fermentation, and harmless, contributing to the efficient use of resources and sustainable agricultural development. However, organic fertilizers contain heavy metals (Cr, Cu, Zn, Cd, Pb, and As) that threaten the safety of agricultural products, soil, and the ecological environment (Zaccone et al., 2010; Muhammad et al., 2020). Many studies have shown that OFS increases the content of heavy metals in soil and agricultural products, depending on its type and application amount of organic fertilizers (Xie et al., 2016; Ning et al., 2017; Xia et al., 2021; Zhang G. B. et al., 2021). Nevertheless, Jia (2017) found that substituting 20% and 40% of the chemical fertilizer with pig manure significantly reduced the content of Cu, Zn, Cd, and Pb in different parts of wheat plants. However, excessive concentrations of heavy metals in agricultural products will adversely impact human health (Muhammad et al., 2020). Thus, for OFS application, the ecological benefits and environmental carrying capacity should be considered. Therefore, studying the effects of different OFSRs on heavy metals in soil and maize grain and assessing their risk will help select a suitable OFSR to avoid polluting the soil and agricultural products.

Our previous field experiments (2017–2018) revealed that OFSRs of 8% and 16% increased maize yield, soil available nutrient content, and economic benefits (He et al., 2021), but its effects on C fractions, middle- and micro-nutrients, and heavy metals were not analyzed further. Therefore, this study conducted pot experiments to investigate the effects of different OFSRs on (1) maize productivity (nutrient absorption, and grain yield and nutrients) and soil fertility (nutrients, C fraction, and CPMI) and (2) heavy metals and their pollution risks in maize grain and soil. This study aimed to determine a suitable substitution ratio to improve maize yield and soil fertility with low pollution risk, providing guidance for scientific fertilization in maize production to promote sustainable agricultural development.

## Materials and methods

### Experimental site and experimental materials

The pot experiment was conducted in the Agricultural Experiment Station of Shihezi University (44°31′N, 86°05′E), Xinjiang, China. The experimental soil was collected from a maize field (0–20 cm) in this experiment station. The soil type was calcareous desert soil (*Calcaric Fluvisol*). Soil basic physicochemical properties were as follows: pH 7.96, SOC 5.52 g kg^−1^, total N 0.65 g kg^−1^, total P 0.83 g kg^−1^, AP 12.38 mg kg^−1^, total K 22.4 g kg^−1^, and AK 149.86 mg kg^−1^.

Chemical N, P, and K fertilizers use urea (N 46.0%), diammonium phosphate (N 18.0% and P_2_O_5_ 46.0%), and potassium sulfate (K_2_O 51.0%), respectively. The commercial organic fertilizer (N 1.78%, P_2_O_5_ 1.96%, and K_2_O 0.53%) was a uniform-sized solid particle made of soybean meal and maize straw, supplied by Zeshang Fertilizer Technology Co., Ltd. (Shihezi, Xinjiang, China). Information on middle- and micro-nutrients and heavy metals content of commercial organic fertilizer and experimental soil is shown in Supplementary Table 1.

### Experimental design

Five treatments were set up as follows: no fertilizer (CK), organic fertilizer substitutes 0% (CF, chemical fertilizer alone), 8% (OF8), 16% (OF16), and 24% (OF24) of the chemical N fertilizer, in which the same amounts of N, P, and K were used for the fertilization treatments. Each treatment consisted of three repetitions, three samples, in a total of 45 pots. Each plastic pot (height 25.0 cm, diameter 20.0 cm) was filled with 10.0 kg of air-dried soil. Six maize seeds (*Zea mays* L. cv. KWS2030) were planted per pot on April 27, 2019. One maize plant remained in each pot when the seedlings reached the two-leaf and one-heart stage. The weighing method strictly controlled 75% of the soil holding water capacity throughout the maize growing season.

The pot fertilization amount of 3.00 g N pot^−1^, 1.80 g P_2_O_5_ pot^−1^, and 0.60 g K_2_O pot^−1^ (Table 1). Before starting the experiment (April 20, 2019), the base fertilizer (i.e., P, K, 40% of N fertilizers, and the commercial organic fertilizer) and 10.0 kg of air-dried soil were mixed well and placed into pots. The remaining 60% N fertilizer (i.e., topdressing) was applied as a nutrient solution to each pot at the booting stage (July 1, 2019).

**Table 1:** Fertilization schemes for different treatments (g pot^−1^).

**Treatment**	**OFSR** **(%)**	**Base fertilizer**						**Topdressing**	**Total nutrient input**		
		**Chemical fertilizer**			**Organic fertilizer**						
		**N**	**P_2_O_5_**	**K_2_O**	**N**	**P_2_O_5_**	**K_2_O**	**N**	**N**	**P_2_O_5_**	**K_2_O**
CK	–	0	0	0	0	0	0	0	0	0	0
CF	0	1.20	1.80	0.60	0	0	0	1.80	3.00	1.80	0.60
OF8	8	0.96	1.54	0.53	0.24	0.26	0.07	1.80	3.00	1.80	0.60
OF16	16	0.72	1.27	0.46	0.48	0.53	0.14	1.80	3.00	1.80	0.60
OF24	24	0.48	1.01	0.39	0.72	0.79	0.21	1.80	3.00	1.80	0.60

Organic fertilizer substitution ratio (OFSR) is the proportion of N input in organic fertilizer to total N input.

### Plant and soil sampling

Before beginning the experiment, the mixed soil samples were air-dried and sieved to determine the soil basic physicochemical properties. Soil samples and maize aboveground plant samples were collected from three pots at the jointing (June 24, 2019), tasselling (July 11, 2019), and harvesting (August 14, 2019) stages.

### Plant and soil sample determination

The dry matter of plant samples (maize aboveground and grain) was determined using the drying method. Plant samples were digested with H_2_SO_4_-H_2_O_2_, and the N, P, and K contents were determined using the Kjeldahl method, molybdenum blue colorimetric method, and flame emission spectrometry, respectively (Bao, 2000).

Soil total N, P, and K were determined using the Kjeldahl method, molybdenum blue colorimetric method, and atomic absorption method, respectively. Soil available nutrients [available nitrogen (AN), phosphorus (AP), and potassium (AK)] were determined using the alkali diffusion method, sodium bicarbonate extraction-molybdenum blue method, and ammonium acetate extraction-atomic absorption method, respectively. Soil organic carbon was determined using the potassium dichromate oxidation method. Refer to Bao (2000) for the above determination methods. Labile organic carbon was determined using oxidation with 333 mmol L^−1^ KMnO_4_ (Loginow et al., 1987). Soil recalcitrant organic C (ROC) was obtained by calculating the difference between SOC and LOC.

The total content of middle-nutrients (Ca, Mg, and S, g kg^−1^), micro-nutrients (Mo, B, Fe, and Mn, mg kg^−1^), and heavy metals (Zn, Cu, As, Cd, Cr, Pb, and Ni, mg kg^−1^) in plants/soil were extracted by the HNO_3_-H_2_O_2_/HCl–HNO_3_-HClO_4_ digestion method (Tessier et al., 1979; Zhang M. et al., 2021). The content of each element was determined using inductively coupled plasma optical emission spectroscopy (Thermo Fisher Scientific Inc., USA). Reference materials for quality control determined soil and plant elements, and the recovery rate was 95–110%.

### Calculation of soil carbon pool indices

Soil C pool indices include the C pool liability index (CLI), C pool index (CPI), and CPMI, calculated as follows (Tang et al., 2020):


(1) $$\begin{array}{rcl} \text{CLI} & = & \frac{{\left(\text{LOC}/\text{ROC}\right)}_{\left(\text{Fertilization treatment}\right)}}{{\left(\text{LOC}/\text{ROC}\right)}_{\left(\text{CK}\right)}} \end{array}$$

(2) $$\begin{array}{rcl} \text{CPI} & = & \frac{\text{SO}{\text{C}}_{\left(\text{Fertilization treatment}\right)}}{\text{SO}{\text{C}}_{\left(\text{CK}\right)}} \end{array}$$

(3) $$\begin{array}{rcl} \text{CPMI} & = & \text{CPI }\times \;\text{CLI}\;\times \;100. \end{array}$$

### Risk assessment of heavy metal pollution

The total content of heavy metals is a common index to assess the degree of heavy metal pollution (Tandy et al., 2009). The combined use of the single factor pollution index (SFPI) and Nemerow comprehensive pollution index (NCPI) could help assess the pollution level of single and multiple heavy metals. Single factor pollution index and NCPI were calculated as follows (Zhang et al., 2017; Wu et al., 2020):


(4) $$\begin{array}{rcl} \text{SFPI} & = & \frac{Ci}{Si} \end{array}$$

(5) $$\begin{array}{rcl} \text{NCPI} & = & \;\sqrt{\frac{{\left(SFPI_{ave}\right)}^{2}+{\left(SFPI_{\max}\right)}^{2}}{2}}. \end{array}$$

where *C*_*i*_ and *S*_*i*_ represent the measured values and limit values of heavy metal content in soil and grain, respectively. *SFPI*_ave_ and *SFPI*_max_ represent the average and maximum values of SFPI, respectively. *S*_*i*_ refers to China's limit standard for soil pollution and food safety (Supplementary Table 2). Information on the pollution levels of NCPI and SFPI is shown in Supplementary text 1.

### Statistical analysis

Data were analyzed using SPSS 21.0 and then plotted with GraphPad Prism 7.0 and Origin 2018. Statistical methods were performed using one-way analysis of variance (ANOVA) and Duncan's multiple comparison test (*p* < 0.05). Measurement data were shown as mean ± standard deviation. Random forest analysis was performed using the package “RandomForest” of R 3.6.1.

## Results

### Maize growth indicators and yield

**Figure 1:**
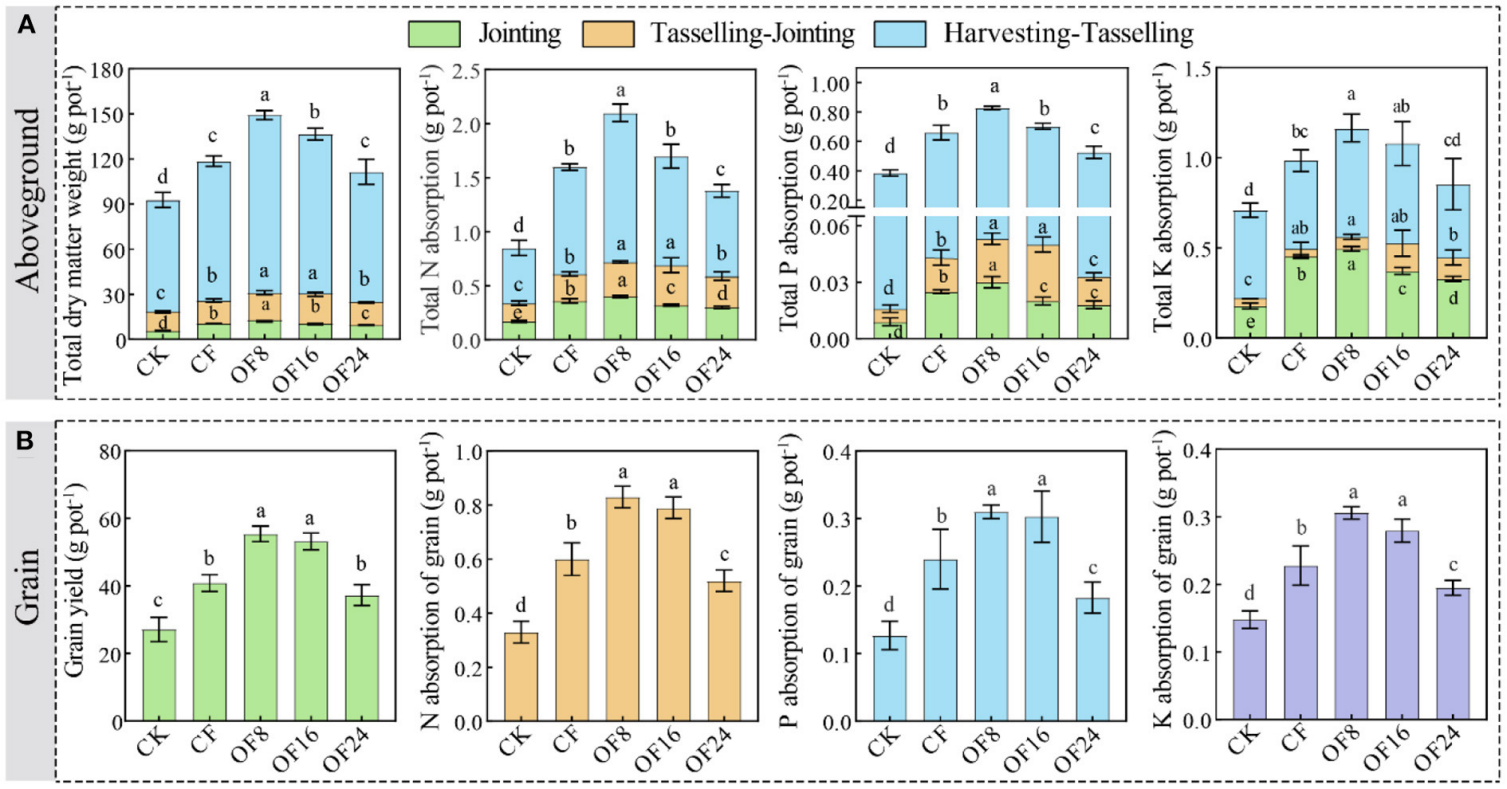
Effects of different treatments on total dry matter, yield, and nutrient absorption of maize aboveground **(A)** and grain **(B)**. The error bars show the standard deviation of the mean of each treatment (*n* = 3). Different lowercase letters represent significant differences at the level of *p* < 0.05.

The total dry matter, N, P, and K absorption of maize aboveground under different treatments showed the following order: OF8 > CF > OF16 > OF24 > CK at the jointing stage and OF8 > OF16 > CF > OF24 > CK at the tasselling and harvesting stages. At harvest, compared with CF, all OFS treatments (OF8, OF16, and OF24) increased the total dry matter, N, P, and K absorption of maize aboveground by 15.04–25.78%, 7.13–31.22%, 7.43–26.15%, and 10.01–18.73%, respectively (Figure 1B). Similarly, the yield, N, P, and K absorption of grain increased by 30.28–35.65% (*p* < 0.05), 31.67–38.89% (*p* < 0.05), 26.39–29.17% (*p* < 0.05), and 22.48–33.87% (*p* < 0.05), respectively (Figure 1B). In addition, with increasing OFSR, each indicator showed a one-variable quadratic function relationship, first increasing and then decreasing (Supplementary Figure 1).

### Soil available nutrients, C fractions, and C pool indices

**Figure 2:**
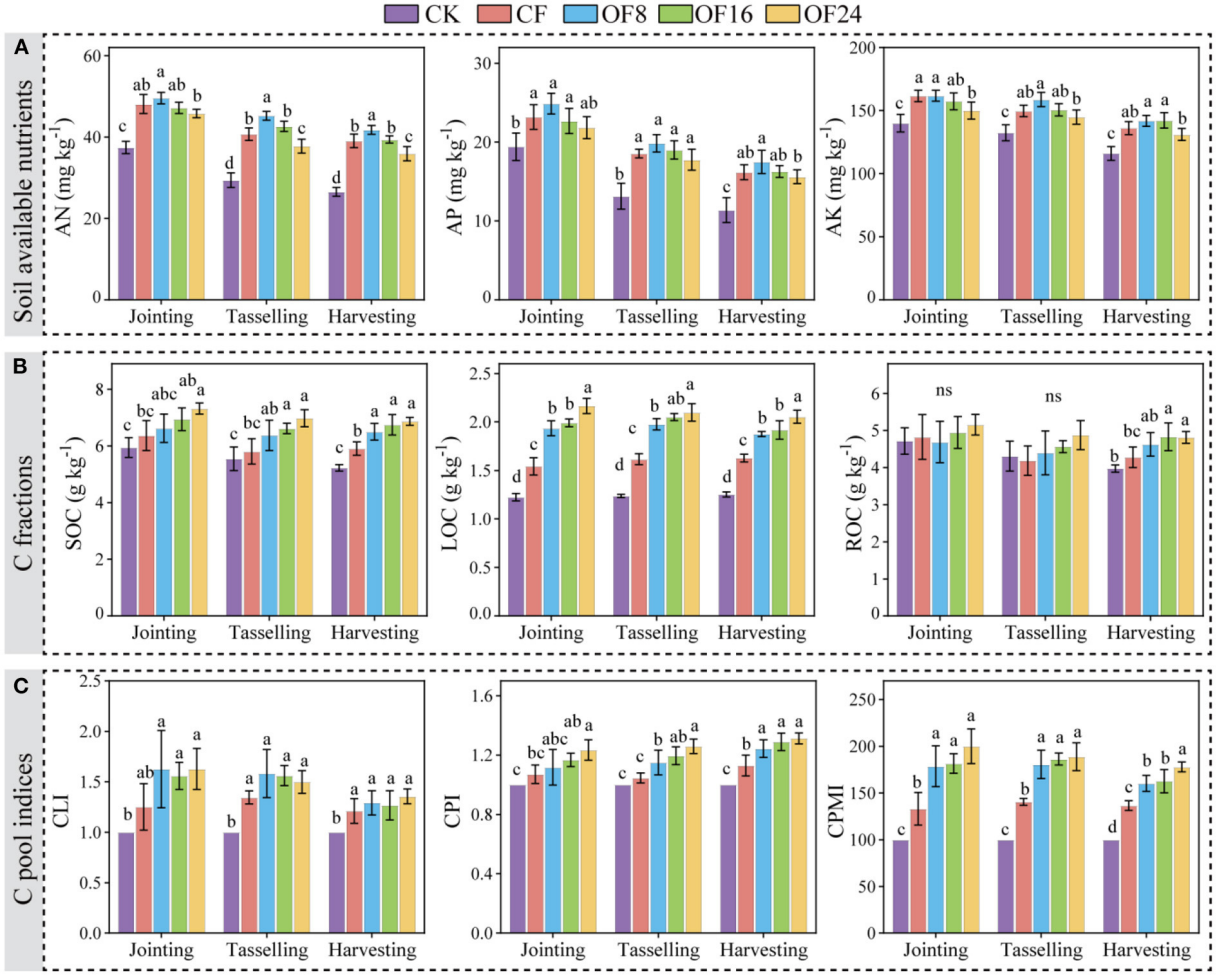
Effects of different treatments on soil available nutrients **(A)**, C fractions **(B)**, and C pool indices **(C)** at jointing, tasselling, and harvesting stages of maize. The error bars show the standard deviation of the mean of each treatment (*n* = 3). Different lowercase letters represent significant differences at the level of *p* < 0.05. Soil available nutrients include available nitrogen (AN), available phosphorus (AP), and soil available potassium (AK). C fractions include soil organic carbon (SOC), recalcitrant organic carbon (ROC), and labile organic carbon (LOC). C pool indices include the C pool liability index (CLI), C pool index (CPI), and C pool management index (CPMI).

At the jointing, tasselling, and harvesting stages, the AN, AP, and AK contents under OFS treatments showed the following order: OF8 > OF16 > OF24. Besides, OF8 was higher than CF, OF24 was apparently lower than CF, and there was no significant difference between OF16 and CF (*p* > 0.05). Compared with CF, OF8 increased AN by 3.04%, 11.15% (*p* < 0.05), and 6.96% (*p* < 0.05), respectively; AP by 7.30%, 7.11%, and 8.05%, respectively; and AK by 0.12%, 6.05%, and 4.17%, respectively (Figure 2A). In addition, soil C fractions and C pool indices of different treatments generally showed the following order: OF24 > OF16 > OF8 > CF > CK. Compared with CF, all OFS treatments (OF8, OF16, and OF24) increased SOC by 3.99–14.99%, 9.78–20.22%, and 10.05–16.26%, respectively; LOC by 25.48–40.38%, 22.25–29.82%, and 15.26–26.20%, respectively; and CPMI by 34.26–50.35% (*p* < 0.05), 28.46– 34.25% (*p* < 0.05), and 17.45–30.31% (*p* < 0.05), respectively (Figures 2B,C).

### Combined analysis of maize growth indicators and soil properties

**Figure 3:**
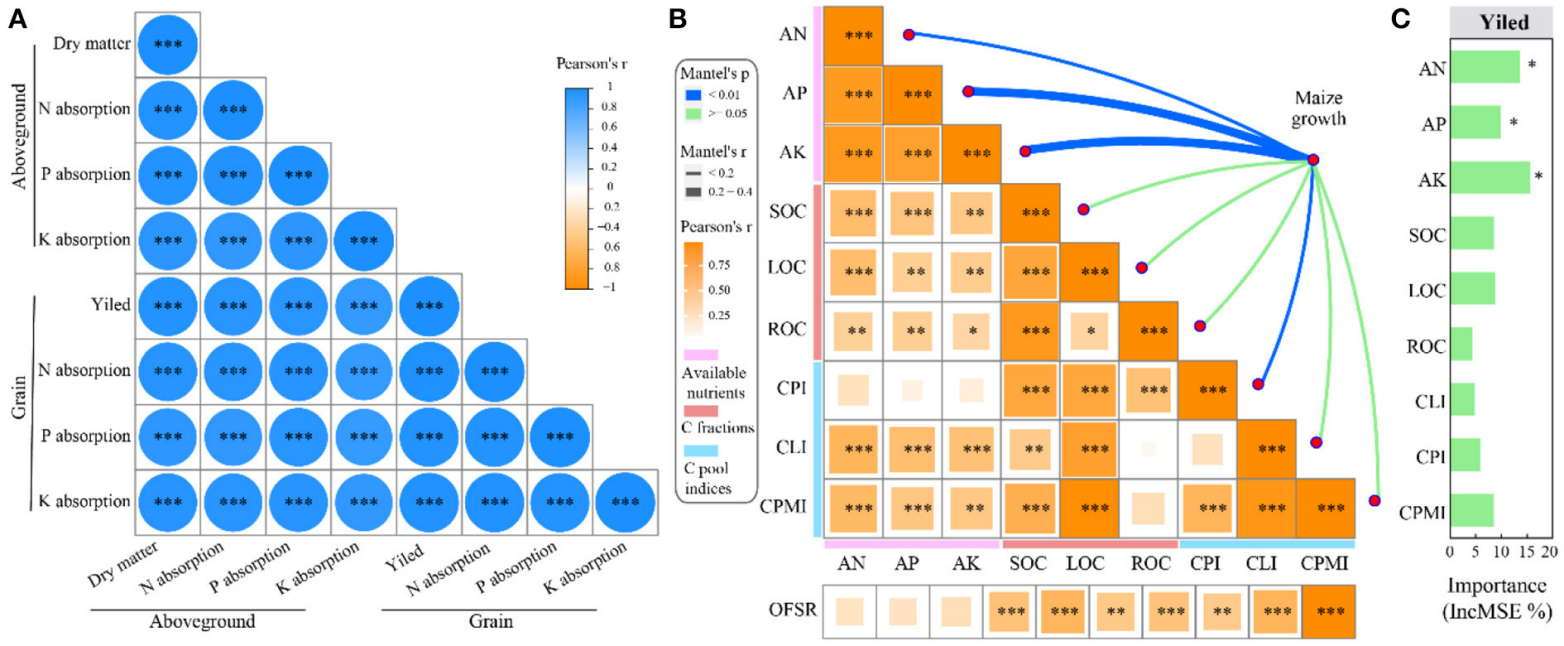
Correlation analysis **(A)** and Mantel analysis **(B)** of maize growth characteristics (total dry matter, N, P, and K absorption of maize aboveground) and soil properties (*n* = 45). Random forest analysis predicts the key soil factors affecting yield **(C)**. *, **, and *** represent significant differences at the level of *p* < 0.05, *p* < 0.01, and *p* < 0.001, respectively. Soil available nutrients include available nitrogen (AN), available phosphorus (AP), and soil available potassium (AK). C fractions include soil organic carbon (SOC), recalcitrant organic carbon (ROC), and labile organic carbon (LOC). C pool indices include the C pool liability index (CLI), C pool index (CPI), and C pool management index (CPMI).

Correlation analysis showed a significant (*p* < 0.001) positive correlation between each maize growth indicators (Figure 3A). There was a significant (*p* < 0.05) positive correlation between soil available nutrients and C fractions. C pool liability index and CPMI showed a significant (*p* < 0.01) positive correlation with soil available nutrients, SOC, and LOC. In addition, OFSR showed a significant (*p* < 0.01) positive correlation with each C fraction and CPI, whereas it did not show a significant correlation with soil available nutrients (Figure 3B).

Mantel test analysis showed that soil available nutrients (AN, AP, and AK) and CPI significantly affected maize growth throughout the growth stage (*p* < 0.01, Mantel'*r* ≥ 0.25, Figure 3B). The random forest model further showed that AN, AP, and AK were key factors affecting yield (*p* < 0.05, Figure 3C).

### Nutrients in maize grain and soil

**Figure 4:**
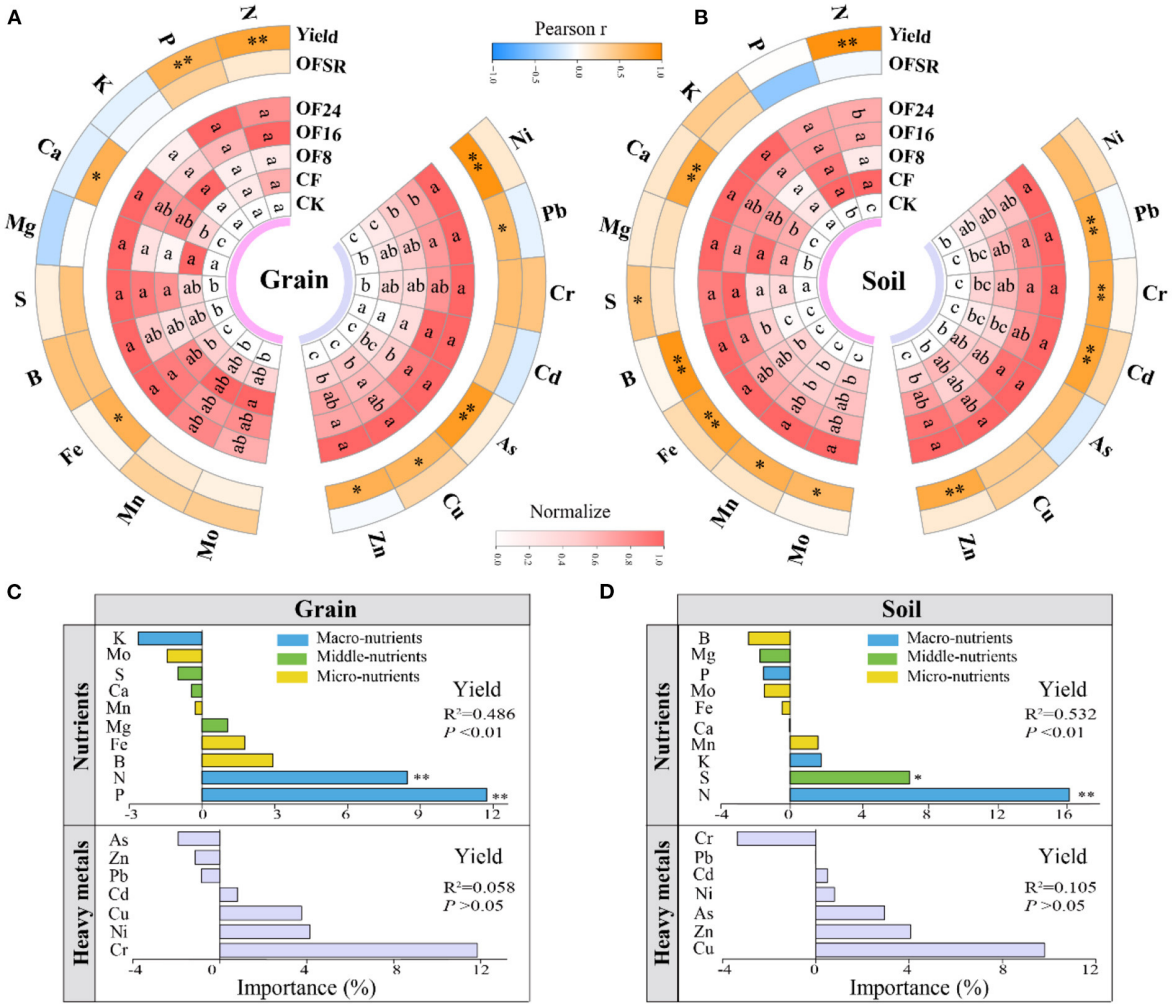
Effects of different treatments on nutrients and heavy metals in maize grain **(A)** and soil **(B)**, as well as their correlation with organic fertilizer substitution ratio (OFSR) and yield. Random forest analysis predicts the key factors affecting yield in maize grain **(C)** and soil **(D)**. In panels **(A,B)**, the degree of shades of red is positively correlated with the normalized value (0–1) of each indicator (*n* = 3). Different lowercase letters represent significant differences at the level of *p* < 0.05. * and ** represent significant differences at the level of *p* < 0.05 and *p* < 0.01, respectively.

All OFS treatments (OF8, OF16, and OF24) apparently increased the content of most middle- and micro-nutrients in maize grain and soil compared with CF but had a weak effect on macro-nutrients content (Figures 4A,B). The content of Ca and Fe in grain and that of Ca, B, Fe, Mn, and Mo in soil showed a significant (*p* < 0.05) positive correlation with OFSR. Compared with CF, all OFS treatments (OF8, OF16, and OF24) increased the content of Ca and Fe in grain by 7.87–13.98% and 13.04–19.10%, respectively; and that of Ca, B, Fe, Mn, and Mo in soil by 1.93–8.80%, 30.17–68.33%, 0.99–7.49%, 0.38–4.59%, and 1.37–14.69%, respectively (Figures 4A,B).

**Figure 5:**
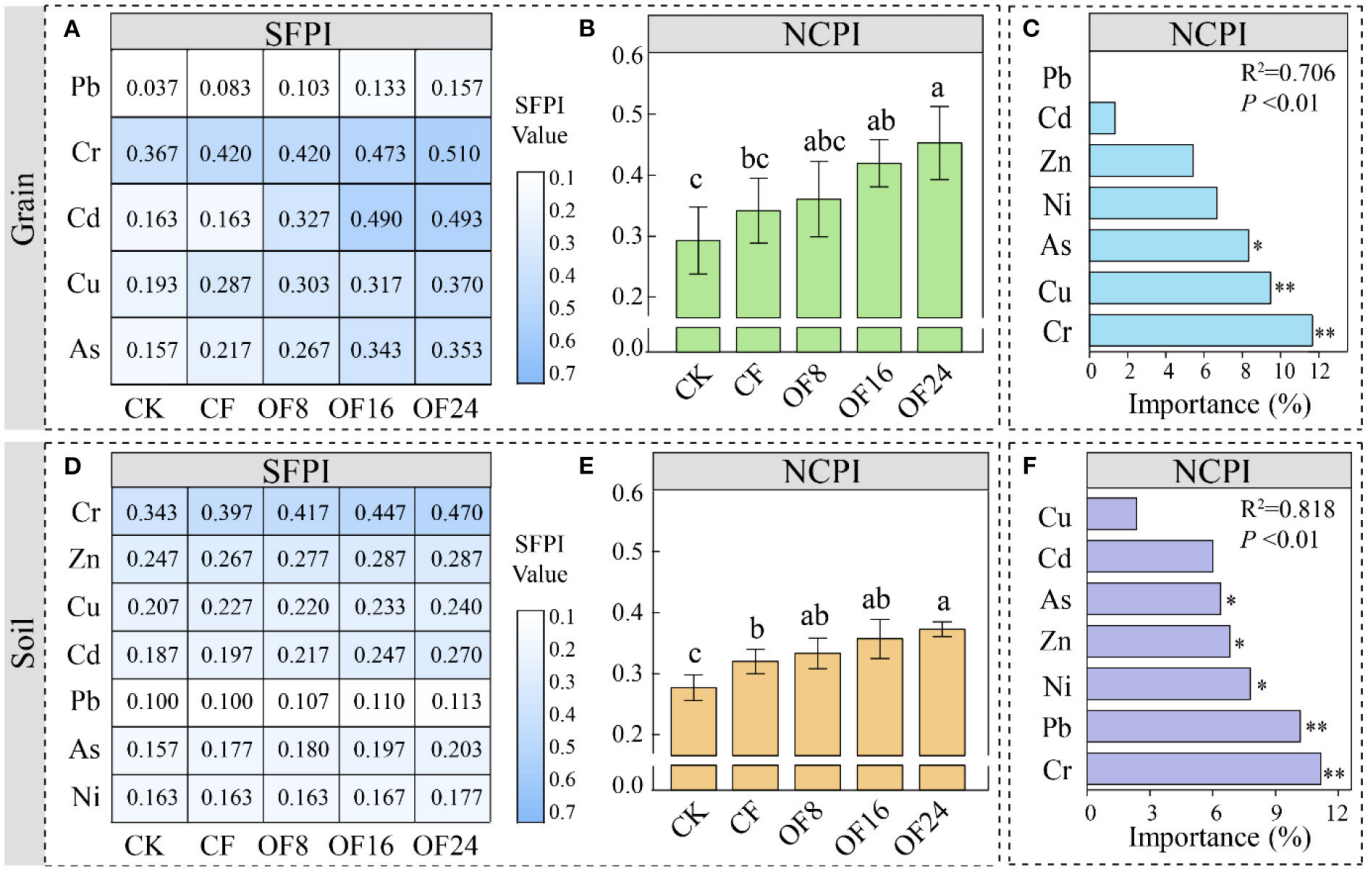
Risk assessment of heavy metal pollution in maize grain **(A,B)** and soil **(D,E)** using the single factor pollution index (SFPI, **A,D**) and Nemerow comprehensive pollution index (NCPI, **B,E**) methods. Random forest analysis predicts the key factors affecting NCPI in maize grain **(C)** and soil **(F)**. In panels **(A,D)**, the degree of shades of blue is positively correlated with the SFPI value of each indicator (*n* = 3). The error bars show the standard deviation of the mean of each treatment (*n* = 3). Different lowercase letters represent significant differences at the level of *p* < 0.05. * and ** represent significant differences at the level of *p* < 0.05 and *p* < 0.01, respectively.

Correlation analysis showed that the nutrient elements in grain and soil had a significant (*p* < 0.05) positive correlation with OFSR (Figures 4A,B); these nutrient elements also showed a positive correlation (*p* < 0.05) with C fractions (Supplementary Figure 2). Furthermore, the content of N and P in grain and that of N and S in soil showed a significant (*p* < 0.05) positive correlation with yield (Figures 4A,B). The random forest model further indicated that the content of N and P in grain and that of N and S in soil were the key factors affecting yield (Figures 5C,D).

### Heavy metals in maize grain and soil

All OFS treatments (OF8, OF16, and OF24) apparently increased the content of heavy metals in maize grain and soil compared with CF (Figures 4A,B) without exceeding their corresponding limit standards in China (Supplementary Table 2). The content of Zn, Cu, As, Pb, and Ni in grain and that of Zn, Cd, Cr, and Pb in soil were significantly (*p* < 0.05) positively correlated with OFSR. Compared with CF, all OFS treatments (OF8, OF16, and OF24) increased the content of Zn, Cu, As, Pb, and Ni in grain by 9.01–20.46%, 4.46–29.06%, 22.69–62.06%, 30.00–90.00%, and 34.35–89.66%, respectively; and that of Zn, Cd, Cr, and Pb in soil by 3.96–7.96%, 10.70–36.79%, 5.18–18.41%, and 5.58–11.20%, respectively (Figures 4A,B).

Correlation analysis showed a significant (*p* < 0.05) positive correlation between the heavy metals in grain and soil and OFSR (Figures 4A,B); these elements also showed a positive (*p* < 0.05) correlation with C fractions (Supplementary Figure 2). However, the correlation analysis and the random forest model showed that heavy metals had no significant effect on yield (Figures 4C,D).

### Risk assessment of heavy metals in maize grain and soil

The SFPI of grain and soil under different treatments ranged from 0.157 to 0.510 and from 0.100 to 0.470, respectively, both at the non-pollution level (all SFPI < 1) (Figures 5A,D and Supplementary text 1). The NCPI of grain and soil under different treatments ranged from 0.292 to 452 and 0.277 to 0.373, respectively, both at the non-pollution level (all NCPI < 0.7) and showed the following order: OF24 > OF16 > OF8 > CF > CF, being higher in grain than in soil (Figures 5B,E and Supplementary text 1). Compared with CF, all OFS treatments (OF8, OF16, and OF24) increased the NCPI of grain and soil by 4.06–16.56% and 2.55–5.57%, respectively. The random forest model showed that Cr, Cu, and As (Cr > Cu > As) in grain and Cr, Pb, Ni, Zn, and As (Cr > Pb > Ni > Zn > As) in soil were the main factors that were responsible for increasing NCPI (Figures 5C,F).

## Discussion

### Effects of different organic fertilizer substitution ratios on yield and soil properties

Recently, OFS has been used to increase yield and soil fertility (Xin et al., 2017; Lv et al., 2020). In this study, OF8 promoted early growth of maize compared with OF18 and OF24, which may be due to the slow efficiency of organic fertilizers (Li et al., 2017; Gai et al., 2018). However, with increasing growth, the nutrients released into the soil by OF8 and OF16 could provide the nutrients required for later growth (tasselling and harvesting stages), thereby increasing yield and nutrient absorption at the harvesting stage (Figure 1). This could be a result of the following: (1) the yield was significantly positively correlated with each maize growth indicator (Figure 3A), suggesting that the treatments promoting growth increased yield; and (2) OF8 and OF16 maintained and increased the content of AN, AP, and AK compared with CF and OF24 and were key indicators significantly affecting maize growth and yield (Figures 2A, 3B), thus promoting growth and increasing yield. Similarly, studies have shown that soil available nutrients are important indicators that ensure crop growth and yield (Ning et al., 2017), and that OFS promotes crop growth to increase yield by increasing soil available nutrients content (Guo et al., 2016; Qaswar et al., 2020). Additionally, there was no significant correlation between soil available nutrients and OFSR; the study conducted by Guo et al. (2016) and the present study found that the yield and OFSR showed a quadratic function relationship that first increased and then decreased (Supplementary Figure 1). These indicated that OF8 and OF16 coordinated the supply of organic and inorganic nutrients during maize growth and maintained the level of soil available nutrients, promoting maize growth and increasing yield. This finding is consistent with that of previous research (Zhang et al., 2016; Geng et al., 2019; He et al., 2021).

Soil organic carbon and CPMI are the major indicators for evaluating changes in soil quality and C pool (Mandal et al., 2020). Application of organic fertilizers increased SOC and LOC content (Li et al., 2017; Gai et al., 2018), whereas the application of chemical fertilizers alone showed the opposite effect (Guo et al., 2016; Li J. et al., 2018). These results are similar to those of the present study, wherein OFS treatments increased the SOC, LOC, and ROC content throughout maize growth (Figure 2B). This finding may be a result of (1) organic fertilizers directly increasing the ROC content after application because they are rich in organic C; (2) organic fertilizers promoting microbial activity and increasing LOC content by accelerating the turnover of organic matter (Li C. X. et al., 2018); and (3) SOC being significantly positively correlated with LOC and ROC, indicating that OFS increased SOC content by increasing LOC and ROC content (Figures 2B, 3B). Other studies have shown that organic fertilizers mainly promote C fixation by increasing ROC content (Yang et al., 2016; Zhang et al., 2016). In addition, CPMI was significantly positively correlated with soil C fractions, suggesting that OFS treatments increase in soil C fraction leads to increased CPMI, augmenting with OFSR (Figures 2B,C, 3B). Similarly, increasing SOC content increases CPMI, and organic fertilizer application effectively increases SOC content and CMPI in a dose-dependent manner (Chen et al., 2018; Li J. et al., 2018), and the improvement was related to the amount applied (Tang et al., 2020; Zhang et al., 2020). In summary, OF8 and OF16 ensured an appropriate maize yield and improved soil fertility by increasing soil available nutrients and C fractions content and CPMI.

### Effects of different organic fertilizer substitution ratios on nutrients in grain and soil

Applying organic fertilizers can improve the content of middle- and micro-nutrients in soil and crops. The present study found that OFS increased the content of middle- and micro-nutrients (Ca, B, Fe, Mn, and Mo) in soil and maize grain but had a negligible effect on N and P content (Figures 4A,B). However, the N in soil and N and P in grain were important factors affecting maize yield (Figures 4C,D). This result is because the amount of organic fertilizer increases with the increase of OFSR, resulting in insufficient nutrient (NPK) supply, affecting maize growth, nutrient absorption, and yield formation. This finding is consistent with that of previous studies (Li et al., 2017; Geng et al., 2019). Moreover, compared with CF, the S application amount of OFS treatments increased by 3.59–10.78% (Supplementary Table 3), which increased soil S content to increase maize yield (Figures 1A, 4B). Similarly, studies found that increasing soil S content or applying S fertilizer can help increase crop yield (Aula et al., 2019; Usmani et al., 2020). Furthermore, applying a combination of organic and chemical fertilizers increases the content of middle- and micro-nutrients in soil and plant, promoting absorption of nutrients by crops and increasing grain yield. In contrast, applying chemical fertilizers alone showed the opposite effect (Zhang et al., 2015).

The content of middle- and micro-nutrients affect maize growth and development and the nutritional quality of the grain, which are closely associated with human health (Zhang et al., 2015; Wajid et al., 2020). This study found that both middle- and micro-nutrients in soil and grain showed a significant positive correlation with the OFSR and soil C fractions (Figures 4A,B), indicating that OFS treatments increased C fractions content and increased the content of some middle- and micro-nutrients. Additionally, Wajid et al. (2020) found that applying organic fertilizers increased the SOC content and affected the transfer of micro-nutrients from soil to the grain. In short, OFS increased the content of middle- and micro-nutrients in maize grain and soil, improving the soil fertility and the mineral nutrient quality of maize grain.

### Effects of different organic fertilizer substitution ratios on heavy metals and their risk assessment in grain and soil

With the improvement of the standard of living of humans, agricultural products and soil safety have been attracting increasing attention (Wang et al., 2016; Adhikari et al., 2020; Ugulu et al., 2020). This study found that OFS treatments increased the content of heavy metals (Cr, Cu, Zn, Cd, Pb, Ni, and As) in grain and soil, which was positively correlated with OFSR (Figures 4A,B). Therefore, OFSR is the main factor affecting the content of heavy metals. Similarly, some studies found that applying organic fertilizer increases the content of heavy metals (Cr, Cu, Zn, Cd, Pb, and As) in the soil and agricultural products (Jia, 2017; Wu et al., 2020). An increase in the soil heavy metal content does not positively affect maize yield, consequently inhibiting its growth and development and thus increasing the content of heavy metals in the grain and the risk of pollution (Figures 4C,D, 5F). Additionally, C fractions were significantly positively correlated with most heavy metals in soil and grain (Supplementary Figure 2), indicating that OFS treatments increase SOC fractions content while also increasing heavy metals content in soil and grain. This may be because SOC contains various functional groups, such as carboxyl, alcohol hydroxyl, and enol hydroxyl, which affect the migration and accumulation of heavy metals in the soil through absorption, chelation, and complexation, thus affecting the accumulation of heavy metals in maize grain (Leszczynska and Kwiatkowska, 2011; Zhao et al., 2014). Studies have found that applying organic fertilizer will reduce the availability of heavy metals in soil. In contrast, long-term chemical fertilizer application also increases the content of heavy metals in soil (Zahra et al., 2010).

Organic fertilizer substitution increased the content of heavy metals in grain and soil may threaten the soil environment and food security. Therefore, evaluating the risk of heavy metal pollution helps guide scientific fertilization. This study found that OFS treatments increased the SFPI and NCPI of soil and grain compared with CF (Figures 5A,B,D,E) but neither reached pollution level. Among them, grain was more susceptible to heavy metal pollution than soil, which was consistent with the findings of Wu et al. (2020). However, Ugulu et al. (2020) found that applying cow manure and poultry manure reduced the risk of heavy metals in wheat grain compared with a single application of chemical fertilizers. Therefore, it is essential to investigate the main elements that affect the increase in NCPI despite the grain and soil heavy metal pollution index not reaching the level that indicated pollution. Meanwhile, this study identified Cr, Cu, and As in grain, and Cr, Pb, Ni, Zn, and As in soil were the main factors contributing to increase in NCPI (Figures 5C,F). Therefore, the long-term application of OFS in maize production requires dynamic monitoring of these heavy metals to prevent the soil quality and agricultural product health from deteriorating.

### Determination of a suitable substitution ratio

**Figure 6:**
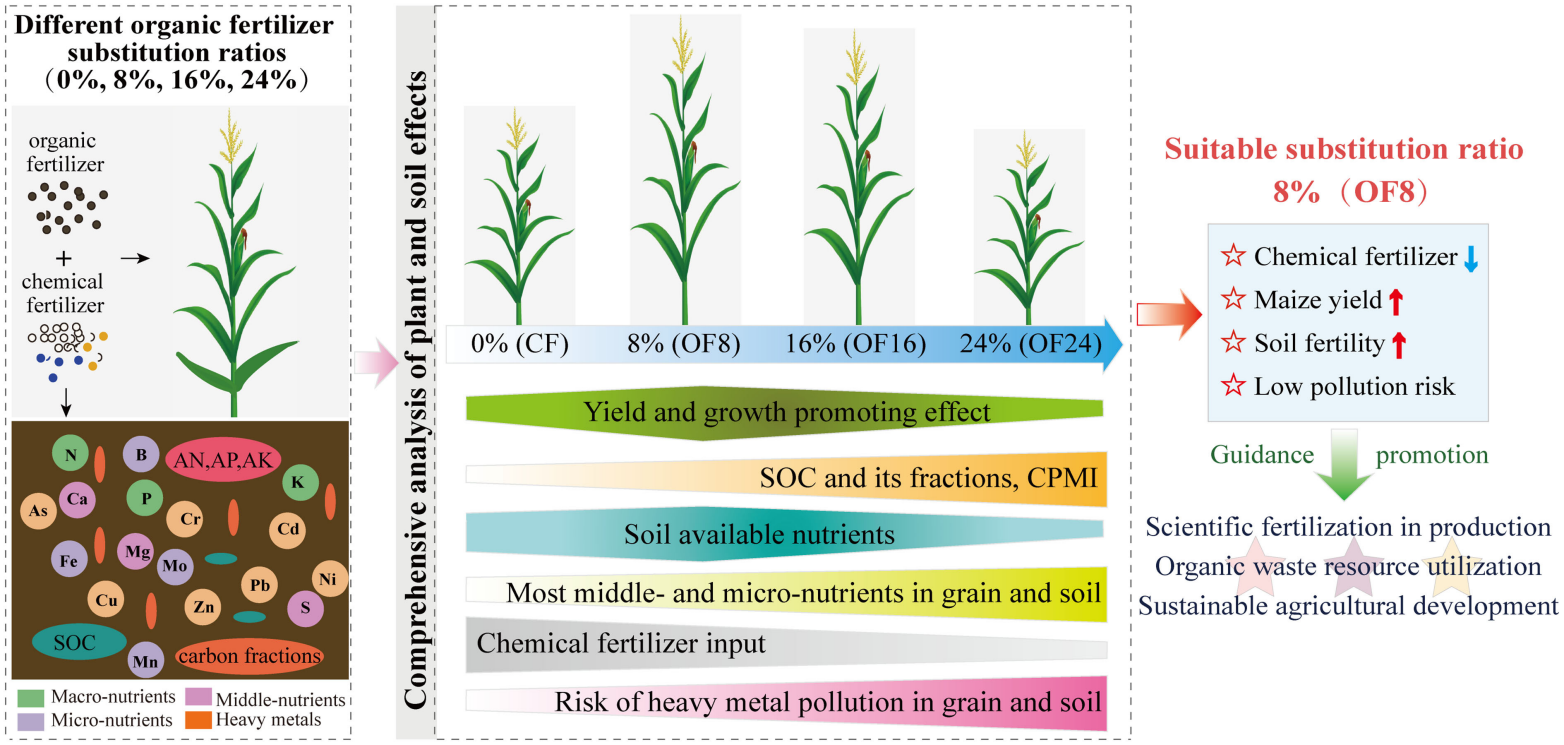
Framework for determination of a suitable substitution ratio.

Whether OFS can become a healthy and sustainable chemical fertilizer reduction measure in agricultural production, its effects on crop yield, soil fertility, and pollution risk need to be considered. Based on the above results, different OFS treatments had different effects on the maize yield, soil fertility, and risk of heavy metal pollution. Therefore, a suitable OFSR should be determined by conducting a comprehensive analysis of soil and plant effects. In this study, compared with CF, all OFS treatments (OF8, OF16, and OF24) (1) improved the mineral nutritional quality of the grain by increasing the content of middle- and micro-nutrients (Figure 4); (2) improved soil fertility by increasing the content of middle- and micro-nutrients in soil and improving soil properties (soil available nutrients, C fractions, and C pool indices) (Figures 2, 3); and (3) increased the content of heavy metals in grain and soil without causing pollution (Figures 4, 5). However, only OF8 coordinated nutrient (NPK) supply throughout the growth period, increasing soil available nutrients (key factor affecting yield), which effectively promoted maize growth and significantly increased yield (Figure 1). Meanwhile, the heavy metal pollution index of grain and soil of OF8 was lower than that of OF16 and OF24 (Figure 5). Accordingly, this study found that a suitable substitution ratio of 8% promoted maize growth, increased maize yield, improved soil fertility, and had a low pollution risk (Figure 6). Compared with previous studies by our (He et al., 2021) and others (Xin et al., 2017; Geng et al., 2019; Lv et al., 2020), this study further determined a sustainable substitution ratio, providing guidance for the scientific application of OFS technology in agricultural production, and promoting the sustainable and healthy development of agriculture.

## Conclusions

The present findings showed that compared with chemical fertilizers alone (CF), organic fertilizer substitution ratio of 8% (OF8) improved soil fertility by increasing soil C fractions and most middle- and micro-nutrients content, and C pool indices, promoting maize growth and increasing yield, and also improving the mineral nutrition quality of grain. Additionally, compared with organic fertilizer substitution ratios of 16% and 24% (OF16 and OF24), OF8 increased maize yield by coordinating nutrient supply throughout the growth period, maintaining a relatively low content of heavy metals in soil and grain; their pollution index (SFPI < 1 and NCPI < 0.7) both at the non-pollution level. Therefore, a suitable substitution ratio of 8% (OF8) increased maize yield and soil fertility while reducing chemical fertilizer application and preventing the risk of heavy metal pollution in the soil and agricultural products. This study provides guidance for the scientific application of OFS technology, promoting the resource utilization, and the healthy and sustainable development of agriculture. Future research work needs to focus on the migration and accumulation of heavy soil metals and their effects on crop growth and soil health under continuous OFS application to ensure soil quality and agricultural safety.

## Data availability statement

The original contributions presented in the study are included in the article/Supplementary material, further inquiries can be directed to the corresponding author/s.

## Author contributions

HH, JL, and ZH planned and designed the study. HH performed experiments and wrote the original draft. MP performed methodology and data analysis. SR, ZH, and JL commented on data interpretation and modified the manuscript. All authors contributed to the study and approved the final manuscript.

## Funding

This research was supported by the National Key Research and Development Program of China (2021YFD1900802 and 2018YFD0200406) and the National Natural Science Foundation of China (31660598, 31360501).

## Conflict of interest

The authors declare that the research was conducted in the absence of any commercial or financial relationships that could be construed as a potential conflict of interest.

## Publisher's note

All claims expressed in this article are solely those of the authors and do not necessarily represent those of their affiliated organizations, or those of the publisher, the editors and the reviewers. Any product that may be evaluated in this article, or claim that may be made by its manufacturer, is not guaranteed or endorsed by the publisher.
