# Supplementary material for: A suitable organic fertilizer substitution ratio could improve maize yield and soil fertility with low pollution risk
Source: Front Plant Sci. 2022 Sep 8;13:988663. doi: 10.3389/fpls.2022.988663 (PMC9511403; doi:10.3389/fpls.2022.988663)
Supplement: Supplementary file 1 [file Data_Sheet_1.docx]

Supplementary Material

# Supplementary Texts

**Supplementary Text 1**

The single factor pollution index (SFPI) and Nemerow comprehensive pollution index (NCPI) were all divided into five pollution levels: ‘SFPI ≤ 1’ is non-pollution, ‘1 < SFPI ≤ 2’ is mild pollution, ‘2 < SFPI ≤ 3’ is moderate pollution, and ‘SFPI > 3’ is heavy pollution. ‘NCPI ≤ 0.7’ is non-pollution; ‘0.7 < NCPI ≤ 1.0’ is alert; ‘1.0 < NCPI ≤ 2.0’ is mild pollution; ‘2.0 < NCPI ≤ 3.0’ is moderate pollution; and ‘NCPI > 3.0’ is heavy pollution (Wu et al., 2020).

# Supplementary Tables

**Supplementary Table 1 |** Content of middle- and micro-nutrients as well as heavy metals in soil and organic fertiliser

| Type | Middle-nutrients  (g kg^-1^) | | | Micro-nutrients  (mg kg^-1^) | | | | Heavy metals  (mg kg^-1^) | | | | | | |
| --- | --- | --- | --- | --- | --- | --- | --- | --- | --- | --- | --- | --- | --- | --- |
|  | Ca | Mg | S | Fe | Mn | B | Mo | Zn | Cu | As | Cd | Cr | Pb | Ni |
| Soil | 28.83 | 12.23 | 0.12 | 2403.67 | 774.53 | 54.90 | 1.70 | 79.15 | 23.96 | 4.41 | 0.11 | 81.61 | 17.23 | 32.13 |
| Organic  fertiliser | 29.74 | 7.79 | 2.45 | 3076.42 | 450.34 | 75.21 | 2.66 | 293.55 | 19.79 | 3.69 | 0.77 | 92.33 | 20.53 | 25.00 |

The heavy metal contents of the soil and organic fertiliser used in this study were all lower than the corresponding limit standards in China. The limit standards refer to the supplementary table Supplementary Table 2.

**Supplementary Table 2 |** Limits of heavy metals in organic fertiliser, soil and grain according to standards in China

| Type | Standard of China | Heavy metals (mg kg^-1^) | | | | | | |
| --- | --- | --- | --- | --- | --- | --- | --- | --- |
|  |  | Zn | Cu | As | Cd | Cr | Pb | Ni |
| Soil (pH > 7) | GB 15618-2018 | 300.0 | 100.0 | 25.0 | 0.6 | 250.0 | 170.0 | 190.0 |
| Organic fertilise | NY 525-2012 | – | – | 15.0 | 3.0 | 150.0 | 50.0 | – |
| Grain | GB 2762-2017 | – | 10.0 | 0.5 | 0.1 | 1.0 | 0.4 | – |

**Supplementary Table 3 |** Application S conditions of different treatments (g pot^–1^)

| Treatment | OFSR  (%) | Potassium sulphate | | Organic fertiliser | | Total nutrient | |
| --- | --- | --- | --- | --- | --- | --- | --- |
|  |  | Application amount | Contains  S | Application amount | Contains  S | S | Compared  with CF |
| CK | - | 0.000 | 0.000 | 0.000 | 0.000 | 0.000 | - |
| CF | 0 | 1.176 | 0.216 | 0.000 | 0.000 | 0.216 | 0.000 |
| OF8 | 8 | 1.039 | 0.191 | 13.483 | 0.033 | 0.224 | 3.593 |
| OF16 | 16 | 0.902 | 0.166 | 26.966 | 0.066 | 0.232 | 7.187 |
| OF24 | 24 | 0.765 | 0.141 | 40.449 | 0.099 | 0.240 | 10.780 |

Note: Potassium sulphate contains 18.4% K_2_O and organic fertiliser contains 0.245% K_2_O.

**Supplementary Table 4 |** Analysis of maize yield and economic benefits of different treatments

| Treatment | Production income | | | Fertilisation input | | Net income  (CNY ha^-1^) |
| --- | --- | --- | --- | --- | --- | --- |
|  | Yield  (kg ha^-1^) | Yield value  (CNY ha^-1^) | Increase income  (CNY ha^-1^) | Fertilisation cost  (CNY ha^-1^) | Increased than CF  (CNY ha^-1^) |  |
| CK | 10.24 ± 0.62 c | - | - | - | - | - |
| CF | 17.05 ± 1.53 b | 23955.11 | - | 2577.15 | - | - |
| OF8 | 18.85 ± 0.85 ab | 26481.65 | 2526.54 | 3973.05 | 1395.90 | 1130.64 |
| OF16 | 19.97 ± 0.23 a | 28055.18 | 4100.07 | 5373.90 | 2796.75 | 1303.32 |
| OF24 | 18.06 ± 2.24 ab | 25368.26 | 1413.15 | 6764.85 | 4187.70 | −2774.55 |

Note: CNY represents Chinese Yuan. Different lowercase letters represent significant differences at the level of *p* < 0.05. References: He et al., 2020.

# Supplementary Figures


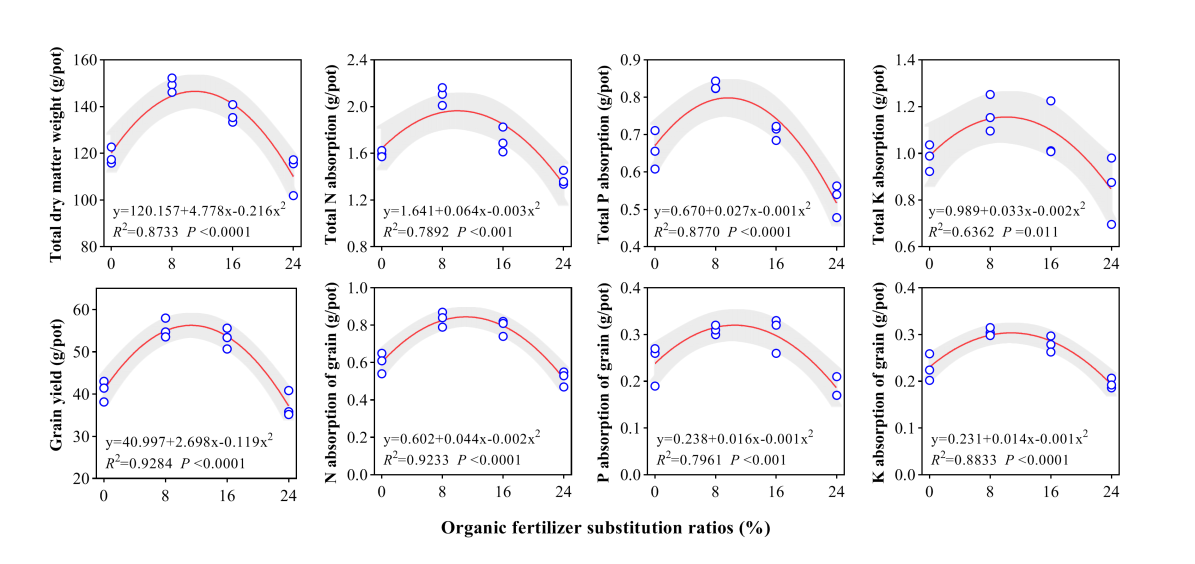


**Supplementary Figure 1 |** Regression analysis of organic fertiliser substitution ratio (OFSR) and maize growth indicators (dry matter, yield, and N, P, K absorption of maize aboveground and grain).


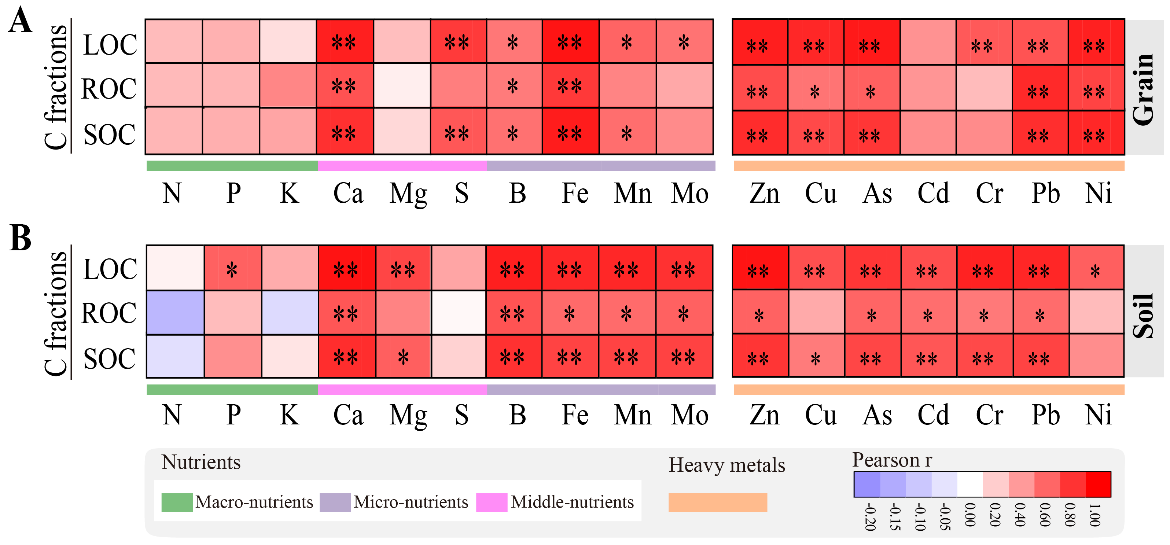


**Supplementary Figure 2** **|** Correlation analysis of nutrients and heavy metals and C fractions in maize grain (**A**) and soil (**B**). * and ** represent significant differences at the level of *p* < 0.05 and *p* < 0.01, respectively. SOC, soil organic carbon; ROC, recalcitrant organic carbon; LOC, labile organic carbon.
